# Supplementary material for: Evaluating empiric antibiotic prescribing for hospitalized children in Mozambique through the introduction of a quarterly syndromic antibiogram: An implementation science protocol
Source: PLoS One. 2024 Aug 9;19(8):e0306511. doi: 10.1371/journal.pone.0306511 (PMC11315278; doi:10.1371/journal.pone.0306511)
Supplement: S1 File — (PDF) [file pone.0306511.s001.pdf]

Training for Lab Technicians (Developing a Syndromic Antibiogram)

- **Each session represents four-hours of in-person training**

Session 1: Introduction to Syndromic Antibiograms

- Importance of Syndromic Antibiograms: Understanding the significance of syndromic antibiograms in antimicrobial stewardship and public health.
- Purpose and Utility: Explaining the purpose of syndromic antibiograms in guiding empirical antimicrobial therapy decisions and monitoring antimicrobial resistance trends.
- Syndromic Approach: Introducing the concept of syndromic approach in antimicrobial susceptibility testing and antibiogram development.
- Syndromic Antibiogram Components: Overview of the components of syndromic antibiograms, including bacterial pathogens, clinical syndromes, and antimicrobial susceptibility profiles.
- Role in Patient Care: Discuss the role of syndromic antibiograms in optimizing patient care by providing timely and targeted antimicrobial therapy.
- Impact on Antimicrobial Stewardship: Highlighting the contribution of syndromic antibiograms to antimicrobial stewardship efforts aimed at reducing antimicrobial resistance and improving patient outcomes.
- Syndromic Antibiogram Development: Brief overview of the process involved in developing syndromic antibiograms, including data collection, analysis, and interpretation.
- Global Guidelines and Standards: Familiarizing with global guidelines and standards, such as those provided by the World Health Organization (WHO), for syndromic antibiogram development and implementation.
- Case Studies: Presenting case studies demonstrating the practical application and benefits of syndromic antibiograms in clinical practice.
- Future Directions: Discuss emerging trends and future directions in syndromic antibiogram research and implementation.
- Q&A Session: Allowing participants to ask questions and seek clarification on the concept and implementation of syndromic antibiograms.

Session 2: Principles of Antimicrobial Susceptibility Testing (AST) Methods

- Introduction to AST: Providing an overview of antimicrobial susceptibility testing (AST) and its importance in guiding antimicrobial therapy decisions.
- AST Techniques: Training on various AST techniques used in clinical microbiology laboratories, including broth dilution, disk diffusion, and automated methods.
- Inoculation Techniques: Demonstrating proper inoculation techniques for preparing bacterial isolates for AST, including inoculum standardization and streaking methods.
- Interpretation of Results: Guiding technicians on the interpretation of AST results, including the determination of minimum inhibitory concentrations (MICs) and zone diameters.
- CLSI Guidelines: Familiarize with the Clinical and Laboratory Standards Institute (CLSI) guidelines for AST interpretation, breakpoints, and reporting standards.
- Quality Control Measures: Emphasizing the importance of quality control measures in AST, such as using reference strains, internal controls, and proficiency testing.

- Troubleshooting: Addressing common issues and challenges encountered during AST, such as reading zone edges, interpreting borderline results, and resolving discrepancies.
- Reporting Standards: Providing guidelines on standardized reporting of AST results, including proper documentation of organism identification, antimicrobial agents tested, and interpretation criteria.
- Hands-on Practice: Offering hands-on practice sessions for technicians to perform AST techniques, interpret results, and report findings accurately.
- Case Studies: Presenting case studies illustrating real-world scenarios and challenges in AST, with emphasis on problem-solving and decision-making.
- Q&A Session: Providing an opportunity for participants to ask questions, share experiences, and seek clarification on AST methods and practices.

Session 3: Software Training

- Introduction to WHONET Software: Overview of the WHONET software and its role in syndromic antibiogram development.
- WHONET Setup and Configuration: Training on setting up and configuring WHONET for antimicrobial susceptibility testing data management.
- Data Entry and Management: Hands-on practice on entering and managing data on microbial isolates, including isolates' identification and antimicrobial susceptibility profiles.
- Analysis and Interpretation: Instruction on analyzing data using WHONET tools and interpreting results to develop syndromic antibiograms.
- Report Generation: Training on generating reports from WHONET, including formatting and customizing reports according to WHO recommendations and CLSI M39 guidelines.
- Quality Assurance: Discussion on ensuring data accuracy, reliability, and adherence to standard protocols in syndromic antibiogram development using WHONET.
- Troubleshooting: Addressing common issues and challenges encountered during WHONET software utilization and providing solutions.
- Best Practices: Sharing best practices and tips for efficient and effective syndromic antibiogram implementation using WHONET.
- Case Studies: Analyzing case studies illustrating successful syndromic antibiogram implementation with WHONET software.
- Q&A Session: Allowing participants to ask questions and clarify doubts regarding WHONET software utilization and implementation in syndromic antibiogram development.

Session 4: Microbial Isolate Data Management

- Introduction to Microbial Isolate Data: Providing an overview of the importance of microbial isolate data in clinical microbiology and antimicrobial stewardship.
- Collection Methods: Training on various methods for collecting microbial isolates from clinical specimens, including swabbing, culturing, and specimen processing techniques.
- Sample Handling: Demonstrating proper handling and storage procedures for microbial isolates to maintain viability and integrity for downstream analysis.
- Identification Techniques: Instruction on microbial identification techniques, including biochemical testing, and automated systems (e.g., MALDI-TOF).

- Antimicrobial Susceptibility Testing (AST): Reviewing the principles and methods of AST for determining the susceptibility profiles of microbial isolates to antimicrobial agents.
- Data Organization: Guiding technicians in organizing microbial isolate data, including patient demographics, specimen source, organism identification, and AST results.
- Data Quality Assurance: Emphasizing the importance of quality control measures in microbial isolate data management, such as regular calibration, validation, and maintenance of laboratory equipment.
- Reporting Standards: Providing guidelines on standardized reporting of microbial isolate data, including proper documentation of organism identification, antimicrobial susceptibility profiles, and interpretation criteria.
- Data Analysis: Introducing basic data analysis techniques for interpreting microbial isolate data, including trend analysis, resistance patterns, and outbreak investigations.
- Hands-on Practice: Offering hands-on practice sessions for technicians to input microbial isolate data into WHONET software, perform data analysis, and generate reports.
- Case Studies: Presenting case studies illustrating real-world scenarios and challenges in microbial isolate data management, with emphasis on problem-solving and decision-making.
- Q&A Session: Providing an opportunity for participants to ask questions, share experiences, and seek clarification on microbial isolate data management practices.

Session 5: Quality Control in Syndromic Antibiogram Development

- Introduction to Quality Control: Providing an overview of the significance of quality control measures in syndromic antibiogram development and their impact on patient care and antimicrobial stewardship.
- Importance of Quality Control: Discussing the importance of quality control in ensuring the accuracy, reliability, and validity of syndromic antibiogram data for clinical decision-making.
- Regulatory Standards: Review regulatory standards and guidelines for quality control in syndromic antibiogram development, including recommendations from organizations such as the World Health Organization (WHO) and the Clinical and Laboratory Standards Institute (CLSI).
- Result Verification: Training on procedures for verifying syndromic antibiogram results, including cross-checking data entries, confirming antimicrobial susceptibility interpretations, and resolving discrepancies.
- Proficiency Testing: Guidance on participating in proficiency testing programs to assess the competency and proficiency of laboratory technicians in performing syndromic antibiogram testing and interpretation.
- Standard Protocols: Emphasizing the importance of following standard protocols and procedures in syndromic antibiogram development, including sample handling, testing methodologies, data analysis, and reporting standards.
- Error Detection and Correction: Strategies for detecting and correcting errors in syndromic antibiogram data, including data entry errors, instrument malfunctions, and specimen
- Documentation Practices: Guidelines for maintaining accurate and detailed documentation of quality control activities, including record-keeping, data logging, and documentation of corrective actions taken.

- Continuous Improvement: Promoting a culture of continuous improvement in syndromic antibiogram development through ongoing monitoring, evaluation, and feedback mechanisms.
- Training and Competency Assessment: Ensuring that laboratory technicians receive adequate training and competency assessment in quality control practices related to syndromic antibiogram development.
- Collaborative Efforts: Encouraging collaboration and communication among laboratory staff, clinicians, epidemiologists, and other stakeholders to address quality control challenges and improve syndromic antibiogram data quality.
- Q&A Session: Providing an opportunity for participants to ask questions, share experiences, and seek clarification on quality control practices in syndromic antibiogram development.
