# Supplementary material for: Evaluating empiric antibiotic prescribing for hospitalized children in Mozambique through the introduction of a quarterly syndromic antibiogram: An implementation science protocol
Source: PLoS One. 2024 Aug 9;19(8):e0306511. doi: 10.1371/journal.pone.0306511 (PMC11315278; doi:10.1371/journal.pone.0306511)
Supplement: S2 File — (PDF) [file pone.0306511.s002.pdf]

Training for Clinicians (Interpreting a Syndromic Antibiogram)

- **Each session represents four-hours of in-person training**

Session 1: Introduction to Syndromic Antibiograms Enhancing Empirical Antibiotic Therapy

- For the session on Introduction to Syndromic Antibiograms, clinicians can be presented with the following subjects:
- Overview of Syndromic Antibiograms: Introduction to the concept of syndromic antibiograms and their significance in clinical practice.
- Explanation of how syndromic antibiograms differ from traditional antibiograms and their benefits in guiding empirical antibiotic therapy.
- Development of Syndromic Antibiograms: Understanding the process of developing syndromic antibiograms, including data collection, analysis, and interpretation.
- Explanation of the sources of data used in syndromic antibiogram development, such as clinical microbiology laboratory results and local antimicrobial resistance patterns.
- Role in Antimicrobial Stewardship: Discussion on the role of syndromic antibiograms in antimicrobial stewardship programs.
- Exploration of how syndromic antibiograms contribute to the rational use of antibiotics and the reduction of antimicrobial resistance.
- Clinical Relevance: Illustration of how syndromic antibiograms aid clinicians in selecting appropriate empiric antibiotic therapy for common infectious syndromes.
- Examples of real-world scenarios where syndromic antibiograms have influenced clinical decision-making and improved patient outcomes.
- Q&A Session: Allowing participants to ask questions and seek clarification on the concept and implementation of syndromic antibiograms.

Session 2: Interpretation of Antibiogram Results

- Understanding Syndromic Antibiogram Reports: Detailed explanation of the components of syndromic antibiogram reports, including susceptibility patterns and resistance profiles.
- Overview of how syndromic antibiogram data is presented, such as graphical representations and numerical summaries.
- Interpreting Susceptibility Patterns: Training on interpreting susceptibility patterns displayed in syndromic antibiograms, including interpretation of minimum inhibitory concentrations (MICs) and zone diameters.
- Discussion on the significance of susceptibility breakpoints and how they inform antibiotic selection.
- Analyzing Resistance Profiles: In-depth examination of resistance profiles depicted in syndromic antibiograms, including identification of resistant pathogens and emerging resistance trends.
- Understanding the implications of multidrug resistance and implications for antibiotic therapy.
- Selecting Appropriate Antibiotics: Guidance on selecting empiric antibiotic therapy based on local resistance data provided in the syndromic antibiogram.

- Discussion on considerations for antibiotic choice, including spectrum of activity, route of administration, and potential adverse effects.
- Q&A Session: Allowing participants to ask questions and clarify doubts regarding the interpretation of syndromic antibiogram.

Session 3: Clinical Decision-Making

- Incorporating Syndromic Antibiogram Recommendations: Guidance on integrating syndromic antibiogram recommendations into clinical decision-making processes.
- Discussion on the importance of considering local resistance data when selecting antibiotic therapy.
- Patient Presentation and Severity of Illness: Training on assessing patient presentation and severity of illness to inform antibiotic selection.
- Understanding the role of syndromic antibiograms in tailoring antibiotic therapy to individual patient needs.
- Antimicrobial Stewardship Principles: Overview of antimicrobial stewardship principles and their application in clinical practice.
- Discussion on how syndromic antibiogram utilization supports antimicrobial stewardship goals, such as promoting appropriate antibiotic use and minimizing antibiotic resistance.

Session 4: Implementation Strategies

- Integrating Syndromic Antibiogram Utilization: Strategies for seamlessly integrating syndromic antibiogram recommendations into clinical workflows.
- Discussion on the benefits of incorporating syndromic antibiogram data into routine clinical practice.
- Effective Order Entry Systems: Training on utilizing order entry systems to access syndromic antibiogram reports and incorporate recommendations into patient care plans.
- Guidance on navigating electronic health record systems to streamline the antibiotic selection process.
- Communication with Laboratory Staff: Importance of effective communication between clinicians and laboratory staff in interpreting and utilizing syndromic antibiogram data.
- Strategies for collaborating with laboratory personnel to ensure accurate reporting and interpretation of antimicrobial susceptibility results.
- Feedback Mechanisms for Quality Improvement: Establishing feedback mechanisms to monitor the effectiveness of syndromic antibiogram utilization and identify areas for improvement.
- Discussion on the role of continuous quality improvement in optimizing antibiotic prescribing practices and patient outcomes.
- Q&A Session: Providing an opportunity for participants to ask questions, share experiences, and seek clarification on Implementation strategies.

Session 5: Case Studies and Practice Scenarios

- Case-Based Learning Exercises: Presenting clinicians with real-life case studies involving patients with suspected bacterial infections.

- Encouraging clinicians to analyze patient scenarios, including clinical presentations, laboratory results, and treatment options.
- Practice Scenarios: Providing clinicians with practice scenarios that simulate common clinical situations encountered in their practice settings.
- Offering opportunities for clinicians to apply syndromic antibiogram interpretation skills to determine appropriate antibiotic therapy for hypothetical patients.
- Real-World Applications: Illustrating the relevance of syndromic antibiogram interpretation in real-world clinical practice.
- Emphasizing the impact of antibiotic stewardship on patient outcomes and public health by using examples from clinical settings.
- Q&A Session: Providing an opportunity for participants to ask questions, share experiences, and seek clarification on case studies and practice scenarios.
