# Supplementary material for: Evaluating empiric antibiotic prescribing for hospitalized children in Mozambique through the introduction of a quarterly syndromic antibiogram: An implementation science protocol
Source: PLoS One. 2024 Aug 9;19(8):e0306511. doi: 10.1371/journal.pone.0306511 (PMC11315278; doi:10.1371/journal.pone.0306511)
Supplement: Attachment — Submitted filename: Reviewer Response 15-03-2024.docx [file pone.0306511.s003.docx]

March 15, 2024

PLOS ONE

Editorial Office

Dear Editors,

We very much appreciate the thoughtful and thorough review of our manuscript. We feel the reviewer´s comments and suggestions have filled in gaps that existed and have strengthened the overall presentation of our work. Below you will find a response to each of the reviewer´s comments. In addition, we have uploaded a new cover letter, a new supplemental file, and a new track change version and clean version of the manuscript.

**Note to Editors:**

*R: We have added an additional author, Dr. Gustavo Amorim, our biostatistician, who was inadvertently left off during our first submission.*

**Response to Reviewers**

**Please ensure that your manuscript meets PLOS ONE's style requirements**

*R: The manuscript has been reformatted per instructions*

**Thank you for stating the following financial disclosure: “Fogarty International Center, National Institute of Alcohol Abuse and Alcoholism of the National Institutes of Health under Award Number D43 TW009745. The content is solely the responsibility of the authors and does not necessarily represent the official views of the National Institutes of Health.” Please state what role the funders took in the study.  If the funders had no role, please state: "The funders had no role in study design, data collection and analysis, decision to publish, or preparation of the manuscript." If this statement is not correct you must amend it as needed. Please include this amended Role of Funder statement in your cover letter; we will change the online submission form on your behalf.**

*R: We have adjusted this statement in our cover letter*

**3. Your ethics statement should only appear in the Methods section of your manuscript. If your ethics statement is written in any section besides the Methods, please delete it from any other section.**

*R: The ethics statement has been placed in the manuscript accordingly*

**Additional Editor Comments:
The authors present a good program of work for the proposed project to improve antibiotic use in the hospital in question. This has potential benefits beyond the hospital and as such invite the authors to revise this submission according to the reviewer comments. I hope this provides a comprehensive review that will improve the project implementation as well as patient outcomes subsequently. In addition to the reviewer comments, kindly provide context on the microbiology support the hospital/program has or will have (internally and or external to the hospital). This should include the tests typically carried out, number of samples processed annually (and by deduction, quarterly), lab turnaround times if available as well as culture positivity rates as most of this project is reliant on the microbiology lab for results to develop the antibiogram.**

*R: Thanks, per the recommendation we have added language about the laboratory in the Study Setting section of the Materials and Methods.*

**Is there designated team member (s) assigned to developing the antibiogram quarterly and what is the proposed timeline for each antibiogram?**

*R: Thank you. The following language has been added to the second sentence of text in the section designated Objective 3: “The first antibiogram will be made just prior to study initiation. Then, in the last two-weeks of each three-month period a new antibiogram will be produced for use during the subsequent three-month period. Existing laboratory technicians will be trained and ultimately responsible for development of each antibiogram, with a goal that this is incorporated into the routine workflow of the lab.”*

**Would each subsequent antibiogram be developed exactly at end of that quarter or at the beginning of the next quarter and how would this affect the proposed timelines?**

*R: Please see our response to the question above.*

**Additionally, a sustainability component is important for success beyond the project. Do the team members have protected time for these project activities, are the activities being carried out routinely or would there need to be additional commitment of time?**

*R: Again, please see our response to the questions above. Our goal is that antibiogram development becomes part of the routine workflow of the lab to ensure sustainability.*

**Reviewer #1: Dear authors,
This is a good study, well done. See my comments below
1. The abstract should clearly state the number of participants/interviews that will be included or the number of prescriptions that will be reviewed.**

*R: We have added language per the reviewer´s recommendations to the Abstract, as well as to the sections for Objective 1 and Objectives 4 and 5.*

**2. The objectives seem to be too many, I hope they can be summarized or some of them combined.**

*R: The authors appreciate the intent of the reviewers comment here, however, the objectives as written are what is in the protocol and what was submitted for ethical approvals. As such we do not intend to alter them here in the manuscript*

**3. Methods: Sample size estimation is missing in the protocol. At this stage, the authors should have and idea at the number of participants/prescriptions that will included in this study. It would be good to add the distribution of healthcare workers at the study site, i.e xx doctors, xx pharmacists, xx nurses, etc, but this may eventually change slightly at the time of data collection.**

*R: Please see our response to Reviewer #1, Question #1 above.*

**4. Limitations of the study: The authors should add some expected limitations to their study.**

*R: Per the reviewer´s recommendations we have added a Limitations section to the paper*

**Reviewer #2: Thank you for the opportunity to review this manuscript. I also want to commend the authors for undertaking this important work in the service of improving outcomes for patients at Hospital Central de Maputo. Best of luck on rolling out this program.

This is a well-written manuscript that aims to fill a gap in the understudied area of implementation of interventions targeting antimicrobial resistance. Recognizing the overall research approach has been reviewed in the course of obtaining funding, my comments are intended to improve the manuscript's and study's impact. I am unable to evaluate details about the antibiogram as an intervention nor the contextual information about antimicrobial resistance, but I can comment on the implementation science methods and approach.**

**Overall comments:
1. The study focuses primarily on implementation of a quarterly syndromic antibiogram as an intervention. But considering the potential results of the knowledge, attitudes, and practice questionnaires and the pre-implementation phase, there may be an equally important need to de-implement current antibiotic prescribing practices. To what extent will your (pre-)implementation findings lead to use of de-implementation strategies and/or draw on de-implementation research and theory?**

*R:We agree with the reviewer that de-implementation of antibiotics is an important potential outcome of the use of our antibiogram. As a result, we have added variables to the Implementation Domain of RE-AIM to capture this information during our study. Please see new Table 1 that has been added.* **2. The role of using DAP to identify implementation strategies is underdeveloped. Do you have any a-priori ideas for which implementation strategies may be helpful and/or have other antimicrobial resistance-related interventions coupled with implementation strategies been specified in the literature? Do you plan to leverage tools like the Expert Recommendations for Implementing Change or processes like Implementation Mapping to select and adapt implementation strategies?**

*R: Please see changes made to the manuscript in the section Study design and conceptual frameworks, Phase one which we hope better describes our use of DAP as language better clarifying the use of a workflow analysis (implementation map).* **3. Align research objectives with the overall study aim and frameworks chosen. Given that the evidence-based intervention being implemented is the quarterly syndromic antibiogram:
a. specific objective 2 should be to elicit barriers/facilitators to implementing the antibiogram (not about rational use of antibiotics). Using a determinant framework can assist with preparing your interview guides for specific objective 2, analyzing your data, and selecting strategies.**

*R:* *Thank you. We have altered the language of Specific Objective #2 to better align with the overall study aim.*

**b. specific objective 5 talks about measuring clinical outcomes and the Effectiveness implementation outcomes appear to be patient-related outcomes, yet the description says there will be no patient outcomes. Please clarify.**

*R: Thank you for pointing out the discrepancy. We have altered the sentence in the Aims and Objectives section, around line 101, to also include clinical outcomes and a measure of effectiveness. Objective number 5 has stayed the same language.* **4. RE-AIM indicators are underspecified. While RE-AIM is an appropriate framework to use to specify what you should be measuring, the manuscript would benefit from further clarity. What are the actual Effectiveness measures of the syndromic antibiogram (and what's the intended effect size)? For Implementation, are there any measures of fidelity (this is alluded to in the figure) and what does consistent delivery of the intervention look like? Please be as specific as possible.**

*R: Thank you. We have added Table 1 to Data Collection Activities Section, under Objectives 4 and 5.*

**5. The manuscript describes using multiple methods, but not mixed methods. While the quantitative and qualitative strands seem appropriate and justified, there doesn't appear to be any blending of the two strands together. Could these data be brought together in a joint visual display? If this is mixed methods and not multiple methods, is this a sequential or a convergent design and which strand is the priority?**

*R: The authors very much appreciated this excellent question. We have provided language in the Study Design section better clarifying that this is a mixed methods study and that results from the different Phases will be used to inform antibiogram implementation strategies.* **6. Introduction needs some context about implementation of antibiograms and/or other interventions targeting antimicrobial resistance. Have others done this and if so, what was their implementation experience? Are there results demonstrating the effectiveness of antibiograms as an evidence-based intervention on the outcomes of interest in this study? If antibiograms are a well-recognized evidence-based intervention and presumably there's a gap in their widespread access or use, then what are the factors that explain why they are not more readily available or used? Is this a quality gap, an access gap, both?**

*R: Thank you. We have added further context to what is now paragraph 4 of the Introduction to better justify use of the antibiogram as a strategy for improvement.*

**Furthermore, it's unclear to a reader unfamiliar with antimicrobial resistance, how innovative this quarterly syndromic antibiogram is. Is the antibiogram innovative because access to this resource is limited in Mozambique? Or is it the specific type of antibiogram being used? Or is it more about how the information is being used to shape prescribing patterns? More clearly delineating how this evidence-based intervention compares to the standard of care and the attendant access/quality gap this program is attempting to address is thus needed.**

*R: We have added language to the second to last paragraph of the Introduction around lines 98-100 to provide better clarity on the fact that there is currently limited information as to the use of antibiograms in Mozambique.* **7. Align figure 1 with description from the 'study design and conceptual frameworks' subsection and specific objectives. The way the figure is drawn appears to contradict the description of the phases and the attendant research questions. There are 3 phases in the description and the separate pre-implementation phase, yet there are only 3 total phases in the figure. Has the pre-implementation phase already been completed and if not, shouldn't that be phase 1? Assessing organizational readiness is mentioned in the figure, but not in the description. Similarly, the bullet points under the RE-AIM evaluation indicators don't align with the description (e.g. feasibility and fidelity are mentioned here but not in the description).**

*R: We have added language into the Study Design and Conceptual Frameworks section so that the text better aligns with the phases presented in Figure 1.*

**8. Specify the recruiting/sampling methods in greater detail.
- specific objective 1: which healthcare professionals will you recruit (all? stratified sample? criterion-based sample, and if so what are the criteria)?**

*R: We have added language into the section for Objective 1 describing the number of health workers and that all will be approached.*

**specific objective 2: what type of purposive sampling will you use? See:** [**https://link.springer.com/article/10.1007/s10488-013-0528-y**](https://nam11.safelinks.protection.outlook.com/?url=https%3A%2F%2Flink.springer.com%2Farticle%2F10.1007%2Fs10488-013-0528-y&data=05%7C02%7Ctmoon2%40tulane.edu%7Ccfcd8e386bf54aca0b6c08dc12028be4%7C9de9818325d94b139fc34de5489c1f3b%7C0%7C0%7C638405049644428569%7CUnknown%7CTWFpbGZsb3d8eyJWIjoiMC4wLjAwMDAiLCJQIjoiV2luMzIiLCJBTiI6Ik1haWwiLCJXVCI6Mn0%3D%7C3000%7C%7C%7C&sdata=wgSW7u%2BIGAULgxAvHnpiswRryyKJPb7AINvaquSBNcc%3D&reserved=0) **as a helpful resource.**

*R: We have added language to Objective 2 to better describe how we will use criterion based purposive sampling and who will be included in the qualitative interviewing.*

**specific objective 4/5: are all of these quantitative or will you use any qualitative data to evaluate things like impact, consistency, or maintenance?**

*R: We hope that the addition of our Table 1 provides better clarity as to the type of data being collected for each of the RE-AIM domains.* **9. The study design is missing some key details.
a. Will there be any comparison relative to the quarterly syndromic antibiogram intervention?**

*R: Thank you for this question. This protocol will serve to conduct a pilot study in a single site in order to inform the development of a broader multi-site study by testing feasibility, refining methods, identifying potential issues, and building the foundation for a successful larger-scale investigation. Please see language added to #3 under the Expected Results of the Research section of the manuscript.*

**b. How will the to-be-determined implementation strategies fit into the design and subsequent analysis? Implementation studies typically compare the relative effectiveness of 1 or more implementation strategies on a particular implementation outcome(s) given a known barrier to implementation.**

*R: To address this question from the reviewer, we first added language to the first paragraph of the Study design and conceptual frameworks, Phase one, that describes how the workflow analysis will take place. Then in paragraphs three and four of the Quantitative Analysis section we have described how this will be analyzed.*

**c. What types of study designs and analytical approaches will be used for measuring impact? Is this a pre-post study or another type of quasi-experimental design comparing the antibiotic prescribing patterns and other effectiveness outcomes?**

*R: Our analysis will include a component of a pre-post analysis to determine impact of implementation of the syndromic antibiogram. See response to prior question.*

**d. Please provide a reference for and specify the qualitative thematic analysis in greater detail. What specific theories will you be engaging with in this part of the analysis? How does your positionality influence the study? Please also mention your intended approach to achieving thematic and/or meaning saturation.**

*R: Per the reviewers recommendations we have re-written the Qualitative Analysis section to address their questions.*

**e. For the quantitative analyses, especially for the effectiveness measures, there should be some discussion about sample size and power calculations.**
*R: Please see updates made to the quantitative analysis section.*

**Line-by-line comments:
line 55: what's the scope of this? Is this globally? In Mozambique?**

*R: We have added the word Globally to the beginning of the sentence*

**line 63: although the implications are important, what does 'reduction in economic productivity' mean? Is this about GDP? I would advise deleting this for brevity.**

*R: We agree and have removed the text.*

**line 64: I would replace 'developing countries' with a more precise choice. Does rising antimicrobial resistance fall along gradients of wealth, geography, healthcare access or inequities, or something else?**

*R: We have replaced “developing countries” with “healthcare settings with limited laboratory capacity”*

**line 76: I would recommend condensing this paragraph, especially the detailed description of different types of antibiograms. This can be replaced with more description about the access/quality gap this program attempts to bridge and more contextual information about implementing antibiograms from other settings.**
*R: The authors have discussed and feel the broader description of the different types of antibiograms is important for the story we are trying to tell. However, we appreciate the recommendation and have added a paragraph describing the gap this program attempts to bridge in the fourth paragraph of the Introduction section.*

**line 120: is there an alternate word choice for strategy? For implementation scientists, this will be confusing to read as the antibiogram is the evidence-based intervention, which is distinct from the implementation strategies used to help implement the antibiogram.**

*R: Thank you, we have removed the word strategy from this sentence*

**line 145: do you have any further information about the training (appropriate for a supplemental file)?**

*R: Per the reviewers request we have added a supplemental file with details about the training curriculum*

**line 146: this workflow analysis seems very promising and fascinating as an implementation strategy. Can you provide any more information?**

*R: We have added language to the first paragraph of the Study design and conceptual frameworks, Phase one, that describes how the workflow analysis will take place.*

**line 191: in the context of an implementation science protocol, this section reads as unnecessary detail and can be deleted in the interest of brevity. However, if this is really critical for a reader who works in this field, then please do keep.**

*R: per the reviewer´s recommendations we have removed this paragraph*

**line 201: sentence is confusingly written. It's also unclear what an 'implementation evaluation approach' is. It would be more clear to just say you are evaluating the implementation of a quarterly syndromic antibiogram. 'Evidence-based' is misleading in this sentence and could be replaced with actionable.**
*R: Per the reviewer´s recommendations we have altered this sentence to be clearer.*

**line 212: although important, the data management section can be reduced in favor of more description of the recruitment and analytical processes.**

*R: The authors feel that the length of the data management section is important for the reader´s understanding of our approaches. As such this section has not been reduced. Through our responses to the other questions raised by the reviewers we feel we have addressed the reviewer´s recommendation here for more description of recruitment and our analytical processes.*

**line 238: how will adherence be measured? This seems critical to both fidelity and effectiveness. Is there a gold standard measure?**

*R: What we are referring to here is the fidelity with which the antibiogram protocols implementation are adhered to. We hope that the addition of Table 1 now better clarifies this question.*

**line 239: depending on the size of your samples, consider an exact test like Fisher's in lieu of a chi-square test.**

*R: We have updated the quantitative analysis section to describe that we will be using both chi-square and Fischer´s Exact test as appropriate. Additional updates have been made to this section to clarify the statistical analyses to be performed*

**line 222: what are the questionnaires you are referring to?**

*R: Here we are referring to a KAP questionnaire we will create for use in this study that was adapted from others in the literature. We have clarified the sentence and added in the references that were also previously cited the section describing Objective 1*

**line 243: the analysis description is underdeveloped. Are you testing a hypothesis? What is the outcome variable of implementation being assessed? Is this a binary outcome of whether or not someone used an antibiogram? The description of the methods implies that the laboratories will be trained in antibiograms so is the question whether or not clinicians order an antibiogram? Or is this outcome based on some other binary measure.**

*R: Please see language added to the last two paragraphs of the Quantitative Analysis section*

**line 245: where do these factors fit into the figure about the phases? Are there any hypotheses about these factors?**

*R: We have added a Table 1 in which we have hoped to better clarify these factors in terms of data source and indicator, or construct used for their measure.*

**line 246: what is LSD?**

*R: We have clarified that LSD stands for Fisher´s least significant difference test.*

**line 255: what's the conceptual framework being used here? Is this being used as an a-priori codebook for deductive coding?**

*R: The term conceptual framework has been removed from this sentence and we have clarified that we will use inductive coding in the text.*

**line 262: are there any implications for adult prescribing? Or in other hospital settings besides the pediatric ward? What about the replicability of the intervention and strategies in other settings (in Mozambique)?**

*R: In response to the reviewer´s recommendation, we have added language to this affect in the second paragraph of the Conclusions.*

**line 272: check word choice of 'withal'**

*R: Thank you, this was a typo and has been removed*

**line 274: this is the first mention that a goal of this study is to reduce hospital stays. Is this an effectiveness measure?**

*R: Reduction in hospital stay is a common measure in the literature of an improved outcome from the use of antibiograms. It is now mentioned earlier in the manuscript, specifically related to text for RE-AIM and the new Table 1 that has been added*

**line 274: I appreciate the diverse dissemination plan described here, but I would instead focus on describing how the quarterly syndromic antibiogram can be used as a learning tool for direct patient care in the context of this study. This is one of the most exciting aspects of this intervention, but it's currently underdeveloped.**

*R: Language has been added to the last paragraph of the conclusions to describe the benefits of the antibiogram as a learning tool for patient care.*

Sincerely,

Darlenne Binta Kenga

University Eduardo Mondlane

Faculty of Medicine

Maputo, Mozambique
