# Supplementary material for: Evaluating empiric antibiotic prescribing for hospitalized children in Mozambique through the introduction of a quarterly syndromic antibiogram: An implementation science protocol
Source: PLoS One. 2024 Aug 9;19(8):e0306511. doi: 10.1371/journal.pone.0306511 (PMC11315278; doi:10.1371/journal.pone.0306511)
Supplement: Attachment — Submitted filename: Reviewer Response 05-03-2024.docx [file pone.0306511.s004.docx]

May 3, 2024

Plos One

Editors

Dear Editors,

Thanks again for the thoughtful review. Please find our responses below

**The** [**PLOS Data policy**](https://journals.plos.org/plosone/s/materials-and-software-sharing) **requires authors to make all data underlying the findings described in their manuscript fully available without restriction, with rare exception, at the time of publication. The data should be provided as part of the manuscript or its supporting information, or deposited to a public repository. For example, in addition to summary statistics, the data points behind means, medians, and variance measures should be available. If there are restrictions on publicly sharing data—e.g. participant privacy or use of data from a third party—those must be specified.**

*R: Thank you. We have added a description of where deidentified data will be stored to the submission portal.*

**Reviewer #2 comments related to Figure 1:**
*R:* *Thank you. We have altered the language in the figure 1.*

**Reviewer #2 comments related to Table 1**

*R: Thank you for your comments. Per recommendations we have moved Reach>Qual Interviews to Implementation and Added Training Data to Adoption. Under Implementation not all are related to Fidelity. We have added track changes to indicate when a measure will be used for feasibility as well.*

**The Data Management section indicates the quant data will come from the KAP questionnaire. Is that the same as the 'surveys and questionnaires' mentioned in the RE-AIM table?**

*R: The survey and questionnaires referred to in the RE-AIM table are different than the KAP survey. In the second paragraph of the Data Management section we have added text to indicate the KAP will occur in the pre-implementation phase and the other survey/questionnaires will occur during the implementation and evaluation phases.*

**Quantitative analysis:
- Your logistic regression proposal looking at these factors and utilization looks fascinating.
- The interrupted time series description indicates you're comparing before and after KAP scores. If there are only 2 time points, then this would be a pre-post analysis and not an interrupted time series. Or are there multiple timepoints?**

R: *Thank you. We have altered the language in the fourth paragraph of the quantitative analysis section to indicate pre-post analysis.*

**Qualitative analysis:
- I don't quite follow the sentence on how deductive codes will be used to formulate main themes. In a thematic analysis, the themes typically result from relationships between codes (regardless of inductive or deductive coding).
- I appreciate the section about Diffusion of Innovations Theory and recognize this may be in response to one of my suggestions about how your qualitative analysis will engage with theory. However, with the clarification that deductive codes will be derived from the RE-AIM framework and the expanded description in Table 1, this now is a bit confusing. I'd now consider removing this paragraph.**

R: *Thank you. We have removed the paragraph around lines 308-311.*

**Limitations:
- I disagree that DAP and RE-AIM lack standardization and that this is a limitation. RE-AIM in particular has extensive resources, standard definitions, checklists, and a guidebook for use on re-aim.org. Regardless, any heterogeneity in their use across different settings and contexts is to be expected. This is even a strength of an implementation evaluation given that you want to understand and harness this context towards identifying implementation strategies that are locally responsive.
- I also disagree that these RE-AIM measures may be subjective or that this is a limitation. Using a framework like RE-AIM gives you an assessment of domains known to be associated with high quality implementation. Adoption, reach, fidelity, acceptability, and feasibility are important intermediates to improving effectiveness outcomes. Furthermore, the rigor of your methods -- e.g., by establishing inter-coder reliability or using repeated measures before and after implementation -- may attenuate this concern. Lastly, even if participants do impart their subjectivity into the data, this can be helpful towards improving implementation.
-I'd strongly consider removing these limitations and instead focus on the limitations of your data collection/analysis methods.**

 R: *Thank you. We have altered the language in the limitations section as requested and to focus more on our data collection/analysis methods.*

Sincerely,

Darlenne Binta Kenga

University Eduardo Mondlane

Faculty of Medicine

Maputo, Mozambique
